# Voice efficiency for different voice qualities combining experimentally derived sound signals and numerical modeling of the vocal tract

**DOI:** 10.3389/fphys.2022.1081622

**Published:** 2022-12-23

**Authors:** Mario Fleischer, Stefanie Rummel, Fiona Stritt, Johannes Fischer, Michael Bock, Matthias Echternach, Bernhard Richter, Louisa Traser

**Affiliations:** ^1^ Department of Audiology and Phoniatrics, Charité—Universitätsmedizin Berlin, Corporate Member of Freie Universität Berlin and Humboldt-Universität zu Berlin, Berlin, Germany; ^2^ Institut Rummel, Frankfurt, Germany; ^3^ Medical Center, Institute of Musicians’ Medicine, University of Freiburg, Freiburg, Germany; ^4^ Faculty of Medicine, University of Freiburg, Freiburg, Germany; ^5^ Medical Center, Department of Radiology, Medical Physics, University of Freiburg, Freiburg, Germany; ^6^ Department of Otorhinolaryngology, Ludwig-Maximilians-Universität München, Division of Phoniatrics and Pediatric Audiology, LMU Klinikum, Munich, Germany

**Keywords:** MRI-data, phonation, finite-element-modeling, sound intensity, voice efficiency, vocal tract resonance, Estill voice training^®^

## Abstract

**Purpose:** Concerning voice efficiency considerations of different singing styles, from western classical singing to contemporary commercial music, only limited data is available to date. This single-subject study attempts to quantify the acoustic sound intensity within the human glottis depending on different vocal tract configurations and vocal fold vibration.

**Methods:** Combining Finite-Element-Models derived from 3D-MRI data, audio recordings, and electroglottography (EGG) we analyzed vocal tract transfer functions, particle velocity and acoustic pressure at the glottis, and EGG-related quantities to evaluate voice efficiency at the glottal level and resonance characteristics of different voice qualities according to Estill Voice Training^®^.

**Results:** Voice qualities Opera and Belting represent highly efficient strategies but apply different vowel strategies and should thus be capable of predominate orchestral sounds. Twang and Belting use similar vowels, but the twang vocal tract configuration enabled the occurrence of anti-resonances and was associated with reduced vocal fold contact but still partially comparable energy transfer from the glottis to the vocal tract. Speech was associated with highly efficient glottal to vocal tract energy transfer, but with the absence of psychoactive strategies makes it more susceptible to noise interference. Falsetto and Sobbing apply less efficiently. Falsetto mainly due to its voice source characteristics, Sobbing due to energy loss in the vocal tract. Thus technical amplification might be appropriate here.

**Conclusion:** Differences exist between voice qualities regarding the sound intensity, caused by different vocal tract morphologies and oscillation characteristics of the vocal folds. The combination of numerical analysis of geometries inside the human body and experimentally determined data outside sheds light on acoustical quantities at the glottal level.

## 1 Introduction

Voice production is regulated by the vibrations of the vocal folds which are driven by the pressure generated by the respiratory system. The generated sound is modified by the resonance properties of the vocal tract (VT) (Titze, 1994). All three components can be controlled independently of each other to a certain extent, but at the same time are also subjected to interactions (Titze, 2008).

Humans can produce a wide variety of sounds characterized by objective acoustic measures such as the fundamental frequency (*f*_*o*_), the sound pressure level and the frequency content but also psychoacoustic properties such as pitch, loudness, vowel, projection, and timbre. In the western classical style of singing, aesthetic aspects are very closely linked to economic aspects of voice production, since it is necessary for the singer to be audible through an orchestra without technical amplification. In contrast, in contemporary commercial music (CCM), a considerably wide range of singing styles is used in an electronic orchestrated surrounding. Here, the comprehensibility of the lyrics and the emotional content of each voice quality plays an important role. In this context, a high degree of voice efficiency might be defined as a voice production mechanism that achieves a high vocal sound pressure level in relation to the energy and bio-mechanical properties that a singer expends. That depends on what the singer wants to achieve. Loud, soft, and neutral sounds, and thus, different degrees of voice efficiency may be produced voluntarily. Still, efficiency plays a crucial role for the singers’ vocal health in addition to the artistically intended sound.

Whether a voice production can be considered “efficient” is determined by the adjustments of the pulmonary, the laryngeal, and the vocal tract system and their interactions. Singers of all genres often sing for several hours, e.g., the CCM industry (Zuim et al., 2021). The vocal effort can lead to increased biomechanical stress and thus to voice disorders when the voice production system is not used efficiently (Czerwonka et al., 2008; Hirosaki et al., 2021).

In western classically trained singing, efficient voice production is associated with the so-called “flow phonation”. Flow phonation shows an increase in the amplitude of vocal fold vibration with a reduction of the glottal resistance and an increase in maximum flow declination rate and thus higher spectral energy (Sundberg, 2015).

In general, efficient voice production amplifies the acoustic power in a region of the sound spectrum sensitively perceived by the auditory system and thus produces a higher vocal sound pressure level. That is known as the singing formant according to Sundberg (1974) (later denoted as singers’ formant cluster (Sundberg, 2015)). An increase in sound pressure level for western classical-style singing is often associated with a low vertical laryngeal position, elongated vocal tract and widening of lip, jaw, and pharynx (Echternach et al., 2014a) as well as narrowing the epilarynx tube (Mainka et al., 2021) especially for male and lower female voices. Yanagisawa et al. (1989) also described an increase in sound pressure level reasoned by an aryepiglottic constriction for different voice production types. A narrow epilarynx tube might also be needed for stable vocal fold vibration when the subglottic pressure increases with sound pressure level (Titze and Story, 1997). This is in contrast to Soprano singing where Mainka et al. (2021) found no correlation between the epilarynx tube volume and the sound pressure level. Generally here, the vocal tract is shorter, the hypopharynx more narrow and the lip opening wider (Köberlein et al., 2021). Sound pressure level can be increased here by aligning the resonance maxima of the vocal tract to partials or the fundamental frequency (known as “tuning”; see Joliveau et al. (2004)). That tuning mechanism was found at frequencies until F6 (1.4 kHz) in classically trained sopranos (Köberlein et al., 2021).

While economic aspects of Western classical voice productions are better understood, the diverse phonation types used in CCM are not investigated in the same way regarding voice efficiency. For such consideration, a definition of sub-classifications is necessary as no widely accepted classification of voice production types exists.

One possible approach is the division according to the adjustment of the vocal tract, which follows one of three fundamentally different strategies according to Titze et al. (2017). A neutral “speech-like” vocal tract configuration (third group) is differentiated from a “megaphone shaped” (second group) vocal tract with a narrowing of the hypopharyngeal part with increased mouth and jaw opening (following the shape of a megaphone or a horn) (Saldias et al., 2019; Echternach et al., 2014a; Saldías et al., 2021). The “megaphone shaped” vocal tract is found in singing qualities called Belting or Twang used on Broadway and in CCM. A similar configuration for very high-pitched female classical singing (e.g., in the third octave > C6, also called “whistle register” is described by Köberlein et al. (2021)). Also, other acoustic characteristics seem to be associated with Belting, e.g., the boost of the second harmonic. Stone et al. (2003) argued that this boost is based on a prolonged closed phase in vocal fold vibration. Schutte and Miller (1993) mentioned the promotion of the second harmonic by the first vocal tract resonance *f*_*R*1_ by raising of *f*_*R*1_ (Titze and Worley, 2009; Sundberg et al., 2012) so that *f*_*o*_ and 2 ⋅ *f*_*o*_ can always be kept below *f*_*R*1_ or that *f*_*R*1_ is tuned to the second harmonic (2 ⋅ *f*_*o*_) (Bourne and Garnier, 2012) (depending on the interpretation by the team of authors).

The first group (“inverted-megaphone” shape) is characterized by a widened oropharynx and hypopharynx coinciding with a moderately opened mouth. This configuration is part of the western classical style of singing. Since the oropharyngeal adjustment exerts a crucial influence on the spoken vowel in differentiating between singing styles, vowel selection is important (Titze et al., 2017).

Concerning efficiency considerations, only limited data is available to date. Very few studies allow intra-individual comparison of these subgroups. In contrast to western style classical singing at the same pitch, for Belting, the vocal tract is described to be shorter and more narrow in the hypopharyngeal area, while the opening of the mouth increased in a single subject experiment in 2D MRI data (Echternach et al., 2014a).

In another single-subject study, Belting had the highest proportion of high-frequency energy in the spectrum, followed by the operatic style, and then by the ordinary speech (Stone et al., 2003). However, there are also substantial similarities in vocal tract configuration between the hypopharyngeal narrowing with high larynx position in Belting and patients with hyper-functional muscle tension dysphonia which, however, exhibited a smaller mouth opening (Saldias et al., 2019).

For a direct comparison of different voice qualities, it would be advantageous to see if a singer could reproducibly retrieve these voice qualities across a large tonal range. While it is uncommon in classical voice training to practice completely different vocal styles equally, this is typical in modern vocal schools like Estill Voice Training^®^. Here singers strive to precisely control individual regulatory parameters of voice production, developing highly differentiated vocal skills. Singers aim to adjust 13 different anatomical parameters individually in different positions, e.g., the tongue position, lateralization of the ventricular folds, narrowing of the epilarynx tube, vertical larynx position, but also the vibrating forms of the vocal folds, and the different possibilities of the glottal closure and opening (Steinhauer et al., 2017). These parameters can be combined to different voice qualities in manifold ways. Seven voice qualities Speech, Falsetto, Sobbing, Nasal, and Oral Twang, Opera, Belting (and their variations) are then explicitly trained during the education program in various pitches according to the specifications originally introduced by Jo Estill as specified in the method section below. The program includes a standardized procedure: passing a standardized vocal exam, including spectral analysis, presentation of the course content, and a written exam on the theoretical background. Based on this concept, it is defensible to study these highly reproducible and different sound production strategies in one professional singer who has reached the highest qualification level in Estill Voice Training^®^. That has the advantage that morphometric differences between subjects cannot falsify the data.

The analysis of vocal tract acoustics is based on different technical methods. Whereas the emitted spectrum of a singer always carries mixed information about the voice source and the resonance properties, it is difficult to analyze the acoustic transfer characteristics of the isolated vocal tract. To overcome this limitation, analyses of vocal tract acoustics are based on the two- or three-dimensional segmentation of the cavities representing the vocal tract. These surface representations are derived from imaging methods such as magnetic resonance imaging (MRI) or computed tomography (CT), which then allows the derivation of the transfer function *via* simulation (Birkholz et al., 2020; Köberlein et al., 2021) or direct acoustic excitation after 3D printing (Fujita and Honda, 2005; Delvaux and Howard, 2014; Echternach et al., 2015; Traser et al., 2017; Fleischer et al., 2018). These techniques allow computation of the location and the level of the vocal tract resonances independently of the voice source. Yet, no measure is available that quantifies the efficiency of the vocal tract configuration in different types of voice production in this respect.

Another approach is the calculation of the impedance of the airways over a frequency range of vocalization. This measure quantifies the degree to which the airway assists or impedes the source in vibration (Titze, 2001) but requires the analysis of its complex frequency response.

The present work aims to compare the acoustic efficiency of the vocal tracts of different voice productions or singing styles defined according to Estill Voice Training^®^ (Steinhauer et al., 2017) in one professionally trained singer for different pitch and vowel conditions.

For this purpose, we used volumetric vocal tract MRI data and audio signals to propose sound intensity at the glottis as a new measure. This is based on the numerically determined acoustic properties and the application of derived filters on a matching voice signal to assess voice efficiency. Based on these calculations, this study aims to quantify differences in the voice efficiency for different voice qualities and investigate correlations between volumetric data of the vocal tract, acoustic data, and electro-glottographic quantities.

Further, we will discuss our findings regarding voice pedagogic implications to raise awareness that specific voice qualities should be used with care. Not only in regards to artistic aspects but also to their efficiency, helping to avoid vocal overload.

## 2 Materials and methods

### 2.1 Data aquisition

The participant in this investigation was a professionally trained female singer and singing teacher, who reached the highest level of Estill Voice Training^®^ certification (Estill Mentor Course Instructor).

This study was approved by the Medical Ethics Committee of the University of Freiburg (206/09). The participant gave informed consent prior to the investigation. This study was performed in accordance with all relevant guidelines and regulations.

Measurements were performed in a 3 T MRI system (PrismaFit, SiemensAG, Munich, Germany). To image the vocal tract a static MRI sequence was applied as described more in detail in Traser et al. (2017). We used a 3D gradient echo with a spatial resolution 1.6 mm×1.6 mm×1.3 mm, echo time 1.6 ms, repetition time 4.76 ms, flip angle 12°, bandwidth 300 Hz/Px, a matrix of 160×119, and a field-of-view of 260 mm×193 mm×62.4  mm. A whole 3D vocal tract scan took 9 s.

The singer was asked to sustain phonation in six different voice qualities (Speech, Falsetto, Sobbing, Oral Twang, Opera, Belting) as defined by Estill Voice Training^®^, for details see Steinhauer et al. (2017):

• Speech: voice quality as used during speaking or in Folk, Jazz or Pop with mid larynx position, wide aryepiglottic sphincter (AES), “thick” vocal folds (similar to modal register in other definitions) and mid position of ventricular folds
• Falsetto: breathy voice quality as used in soft/high harmonies of A cappella groups, early music, Folk, Jazz with mid larynx position, wide AES, “stiff” (abducted) vocal folds and mid position of ventricular folds. As defined for this study, Falsetto is not identical to the category used for register.
• Sobbing: dark voice quality similar to mourning as used in operatic pianissimo and Blues with low larynx position, wide AES, “thin” vocal folds (similar to head register in other definitions) and retracted ventricular folds
• Oral Twang: bright voice quality as used in country music with high larynx position, narrow AES, “thin” vocal folds and retracted ventricular folds
• Opera: voice quality as heard in Opera/Oratorio with low larynx position, narrow AES, “thin” and sometimes “thick” vocal folds and retracted ventricular folds.
• Belting: voice quality typically used in American musical theatre and Gospel music whenever it is loud or dramatic with a high larynx position, narrow AES and “thick” vocal folds and retracted ventricular folds

Voice qualities that require a pronounced opening of the velum, were not included (e.g., nasalized twang). Here according to Havel et al. (2021) a segmentation of the whole nasal system would be necessary.

The participant was asked to sing in a supine body position and advised to choose the loudness according to the typical medium loudness for the respective voice quality. The tasks were first performed in A♭3 (G^*♯*^3, 208 Hz) on vowel /aː/ followed by A♭4 (G^*♯*^4, 415 Hz) vowel /aː/ and vowel /iː/. To support the singer in the performance as intended after Estill Voice Training^®^, we simultaneously recorded the voice using an MRI-compatible microphone (FOMRI I; MR Confon Magdeburg, Germany) which was displayed live as a spectrogram of the voice for real-time feedback on a monitor. The intense sound produced by the MRI and the acoustic environment preclude high-quality recordings during scanning. However, between and during the tasks, viewing the spectrogram was very helpful for the singer to maintain singing quality. Repetition of the tasks was possible.

In a second measurement, we asked the participant to perform the same tasks in a sound-treated room for high-quality audio and electroglottographic recordings (EGG, Glottal enterprises, Syracuse NY, United States, EG2-PCX2). In order to ensure the highest possible comparability with the MRI images, these recordings were also performed in the supine position. Before recording, the sound pressure level (*L*_*eq*_) calibration was performed by carefully matching a calibrated front microphone (calibrated by an 94 dB SPL calibrator device) to the used headset microphone (DPA 4066-OC-A-F00-LH beige, 4066 CORE Omni Headset, DPA Microphones, Inc., Longmont, CO, United States), for recording (see Ternström et al. (2018)). We placed the headset microphone 7 cm lateral of the center of the lips. For the implemented filtering technique (as described below), we extracted a part of stable phonation of the audio recording using Adobe Audition (CS6, Version 5.0, www.adobe.com). For plausibility purposes, we used manual inverse-filtering techniques to determine vocal tract resonance frequencies in the audio signal using the DeCap software (DeCap, v3.0.0.1, Granqvist et al. (2003)).

### 2.2 3D vocal tract model creation

The segmentation of MRI data, implementation of teeth models and preparation of the model for simulation was performed as described more in detail in Birkholz et al. (2020). Firstly, we interpolated the MRI data to a uniform voxel length of 0.35 mm. We further merged the boundary models of the maxilla and mandible (scanned *via* iTero scanning device (Aligne Technology, San Jose, CA), see Traser et al. (2017)) with the MRI data of the vocal tract shape. We positioned the meshes of the mandible and maxilla according to anatomic landmarks (e.g., tooth roots, palate shape, respecting the MRI data). Finally, we set the resulting voxels of the tooth models to a constant mid-level gray value. For all pre-processing steps we used the Software 3D Slicer (Fedorov et al. (2012), www.slicer.org).

High-resolution data from these pre-processing steps were the base for semi-automatical segmentations *via* ITKSNAP 3.8.0 (Yushkevich et al. (2006), http://www.itksnap.org). Here, we used the implemented active contour method to derive surface models for all MRI-data. Three physicians independently checked all surface models (co-authors SR, SF and LT), including manual fine control in unison (Figure 2).

**FIGURE 1:**
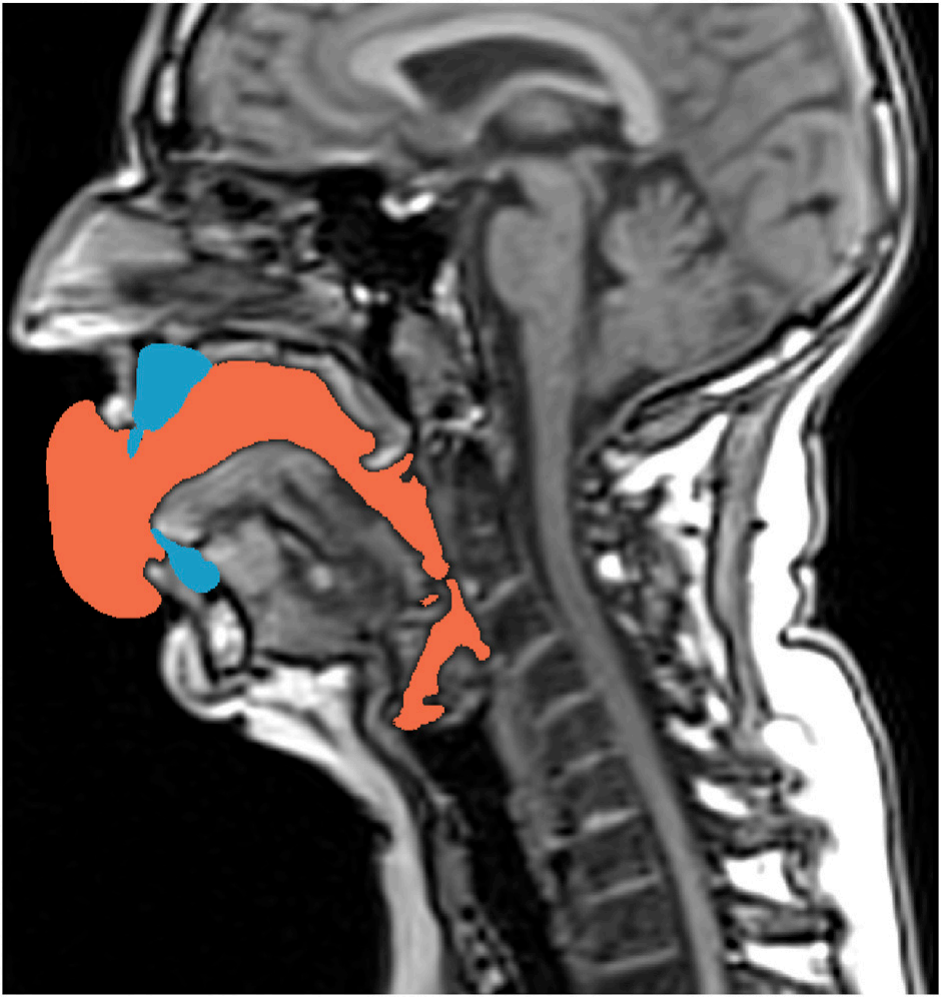
Exemplarily shown for Belting /aː/ (A♭3), the mid-sagittal slice of the MRI stack shows the air-filled cavities in black color. Additionally, the post-processed teeth models (blue) and the finally derived segmentation of the vocal tract cavity used in the FE calculation (orange)—considering the teeth not shown in the primary MRI data–are overlayed.

The result of the segmentation process were surface representations of the air-filled, laryngo-, hypo-, and oro-pharyngeal cavities with an extension into the free space in front of the open mouth (see Figure 1). The bubble in front of the mouth opening was manually adapted using Meshmixer 3.5.474 (Autodesk® Inc. 2017; https://www.meshmixer.com) to a uniform bubble.

### 2.3 Derivation of volumetric data

**FIGURE 2:**
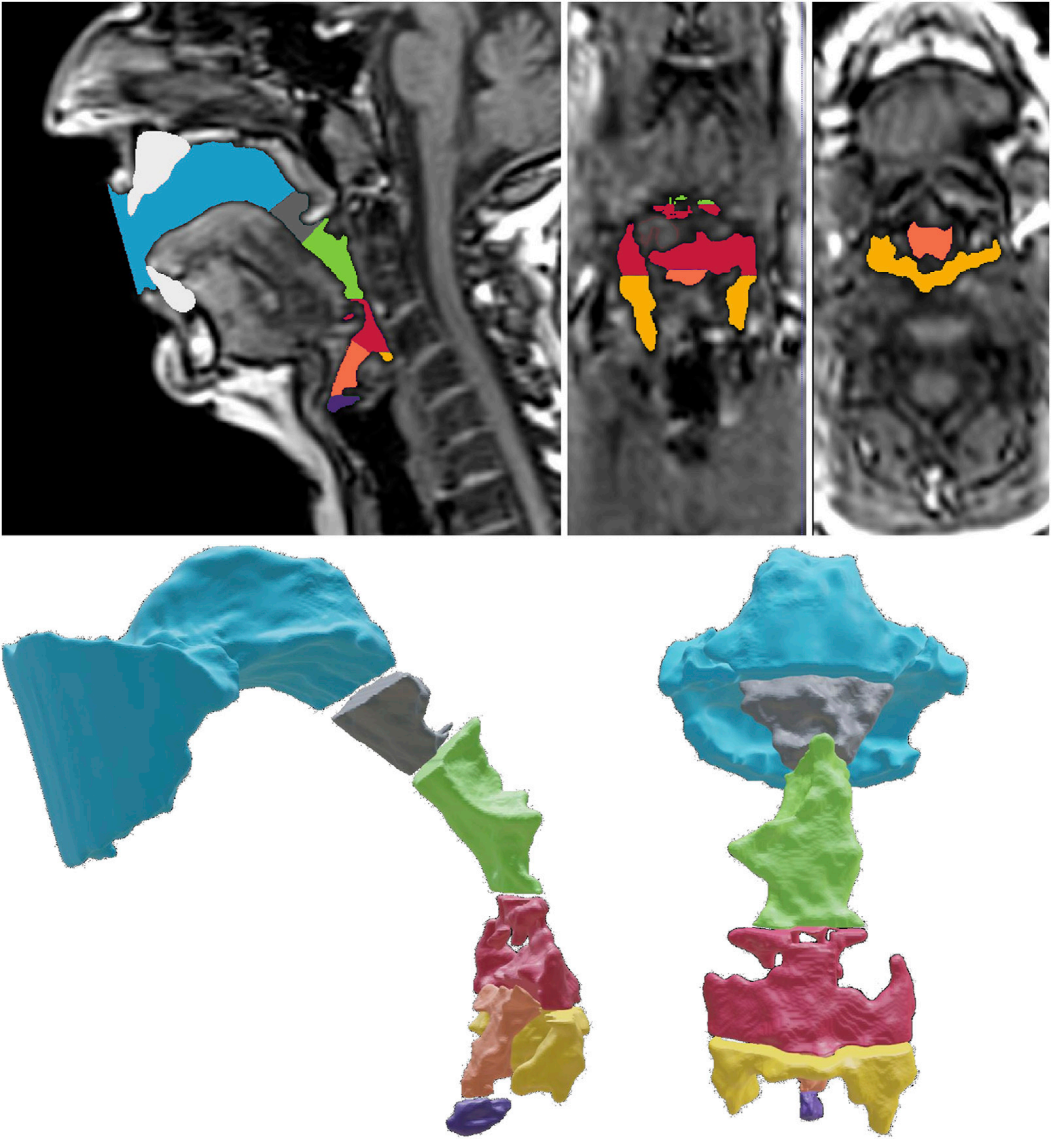
Definition of seven sub-volumes needed for the volumetric analysis of the sub-volumes (exemplarily shown for Belting /aː/ (A♭3). The upper row shows the mid-sagittal, coronal, and the transversal plane of the MRI data and the segmented cavities. The lower row shows the exploded view of the sub-volumes in the sagittal and coronal view. The blue color is used to highlight the oropharynx front (OPf), and the oropharynx rear (OPR) is shown in gray. The upper hypopharynx (HPu) is highlighted in green, whereas the lower hypopharynx (HPl) is highlighted in red. Finally, the epilarynx tube (ET), *Sinus piriformis* (SP), and ventricular volume (VV) are shown in orange, yellow, and purple, respectively.

To derive geometrical data from each vocal tract, we seperated each model into seven sub-volumes. The dissection procedure follows anatomical landmarks according to the following definition (see Figure 2).

We defined the frontal part of the oropharynx as the volume restricted by the tangential plane of the lips and the back of the nasal septum (Oropharynx front, OPf). Connected to OPf, we defined the rear part of the oropharynx (Oropharynx rear, OPr) following downstream up to the lowest point of the uvula. The hypopharynx is connected to OPr and limited by the lowest edge of the aryepiglottic fold. However, we split the hypopharynx into an upper (HPu) and a lower section (HPl) at the highest point of the epiglottis. Further, we defined the epilarynx tube (ET) as a sub-volume restricted by the aryepiglottic fold and the upper edge of the *plicae vestibulares*. The ventricular volume (VV) was defined as the space between the upper edge of the *plicae vestibulares* up to the vocal fold level (thus representing the *sinus morgagni*). The *sinus piriformis* (SP) are the air-filled spaces caudal to the lowest edge of the aryepiglottic fold. Further, we defined the sum of oropharynx front and rear as the whole oropharyngeal volume OP, whereas we defined the sum of all other volumes as the hypopharyngeal (and laryngeal) volume (HP). The sum of OP and HP represents the bulk volume V of the vocal tract.

The length L, starting at the upper limit of the vocal folds up to the lateral opening of the lips, of the vocal tracts was derived as described in Echternach et al. (2015).

### 2.4 Vocal tract transfer function

**FIGURE 3:**
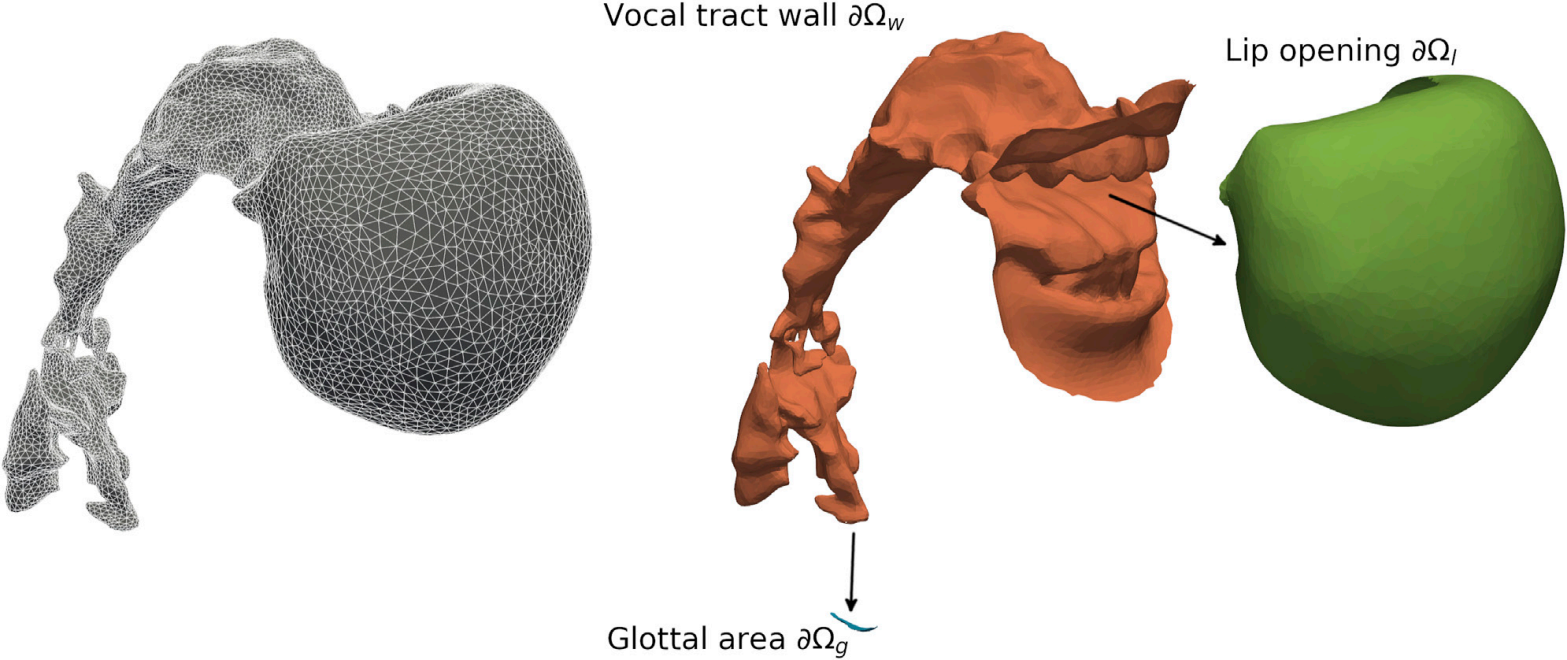
Mesh of the Finite-Element-Model (domain Ω, left in gray) and exploded view (right) to depict the patches for the lip opening (*∂*Ω_*l*_, green), the vocal tract wall (*∂*Ω_*w*_, orange), and the glottal area (*∂*Ω_*g*_, blue) for Belting /aː/ (A♭3).

The surface representations of the vocal tract for each vowel and voice quality were post-processed using the method presented in Birkholz et al. (2020). In brief, we smoothed each stl-mesh and reduced the number of facets in Meshlab (www.meshlab.net). Further, we split all stl-files into parts for the glottal entry, the lip opening, and the vocal tract wall using Blender (www.blender.org) (see Figure 3). Finally, we created volume meshes (msh-files) using GMSH (www.gmsh.info) to be converted into xdmf-files.

We analyzed the xdmf-files within the open-source Finite-Element-Software dolfinx (https://jorgensd.github.io/dolfinx-tutorial/). The mean element size for all models was between 1.43 and 2.6 mm. That results in a spatial resolution of about 13 to about 24 elements with linear shape functions per wavelength at 10 kHz. Therefore, for all models, the spatial resolution ensures high precision of the numerical analysis within the chosen frequency range of up to 10 kHz.

We analyzed the acoustic transfer characteristics for all models by solving the Helmholtz equation

$$-\left(\kappa^{2}+\nabla^{2}\right)\;p\left(\vec{x},\;\omega \right)=0,\;\forall \vec{x}\in \Omega$$ (1)

for the unknown complex-valued scalar pressure *p* at all positions $\vec{x}$ within the segmented volume Ω. More precisely, the domain Ω contains the air-filled cavity bordered by the areas representing the glottal opening (*∂Ω*_*g*_), the vocal tract wall (*∂*Ω_*w*_), and the lip opening (*∂Ω*_*l*_) according to Figure 3. *κ* = *ω*/*c* is the wavenumber with the angular frequency *ω* = 2*πf*. We varied the excitation frequency *f* from 0 up to 10 kHz in steps of 10 Hz. The speed of sound, *c*, was set to 353 m/s for all models, corresponding to a value at a temperature of 37°C. At the glottal entry *∂Ω*_*g*_, we applied the Neumann-boundary condition

$$\nabla p\left(\vec{x},\;\omega \right)\cdot \vec{n}=-j\omega \varrho_{0}V_{0}\;\forall \vec{x}\in \partial \Omega_{g}$$ (2)

with a constant particle velocity *V*_0_ and a constant density *ϱ*_0_ = 1.13 kg/m^3^. $\vec{n}$ is the pointing outside normal vector of unit-length perpendicular to the surface. At the vocal tract wall *∂*Ω_*w*_, we applied Robin-boundary conditions

$$\nabla p\left(\vec{x},\;\omega \right)\cdot \vec{n}=-j\kappa \frac{\varrho_{0}c}{Z_{w}}p\left(\vec{x},\;\omega \right)\;\forall \vec{x}\in \partial \Omega_{w}$$ (3)

with a real-valued impedance *Z*_*w*_ = 200 ⋅ *ϱ*_0_ *c* which is equivalent to a boundary admittance coefficient *μ* = 0.005 (Arnela et al., 2013; Arnela and Guasch, 2014; Zappi et al., 2016). To incorporate the spherical shape of the lip facets *∂*Ω_*l*_ and to guarantee absorption of outgoing spherical waves, we applied Robin-boundary conditions

$$\nabla p\left(\vec{x},\;\omega \right)\cdot \vec{n}=-j\kappa \frac{\varrho_{0}c}{Z_{l}}p\left(\vec{x},\;\omega \right)\;\forall \vec{x}\in \partial \Omega_{l}$$ (4)

with a mathematical expression for the radiation impedance *Z*_*l*_ according to Birkholz et al. (2020). In contrast to Birkholz et al. (2020), we considered a lip opening mimicking a bubble to avoid any overlap with internal structures such as the teeth. For each model, we calculated first the complex-valued transfer function

$$H_{g}^{l}\left(\omega \right)=\frac{p_{l}\left(\omega \right)}{V_{0}}$$ (5)

for *p*_*l*_(*ω*) central at *∂Ω*_*l*_. We derived

$$H_{g}^{g}\left(\omega \right)=\frac{p_{g}\left(\omega \right)}{V_{0}}$$ (6)

for *p*_*g*_(*ω*), central at *∂Ω*_*g*_.

Using the glottal area *A*_*g*_ and the lip opening area *A*_*l*_, we computed the volume velocity transfer function (VVTF)

$$V_{g}^{l}\left(\omega \right)=\frac{p_{l}\cdot A_{l}\left(\omega \right)}{Z_{l}\cdot V_{0}\cdot A_{g}}.$$ (7)

All stl-models, the subsequently derived files, the result files, and analysis scripts are available at Fleischer et al. (2022).

### 2.5 Filter approximation

To calculate acoustic quantities (particle velocity $v_{g}^{\text{sound}}\left(t\right)$ and the acoustic pressure $p_{g}^{\text{sound}}\left(t\right)$ ) at the glottis (that cannot be determined experimentally) based on experimentally derived sound records *p* ^sound^(*t*) at the lips, we approximated the *via* FEM numerically computed transfer functions $H_{g}^{l}\left(\omega \right)$ and $H_{g}^{g}\left(\omega \right)$ by the infinite-response-filters (IIR) ${\hat{H}}_{g}^{l}$ and ${\hat{H}}_{g}^{g}$ of the length of 256 samples, respectively, using the LSIIR-class in the PyDynamic-package (v2.3.0, Eichstädt et al. (2017)). Before computing the IIR, we filtered *p* ^sound^(*t*) with a high-pass Butterworth-filter of 10th order with a corner frequency of 0.8 ⋅ *f*_0_, and downsampled *p* ^sound^(*t*) to the two-fold maximum frequency of 10 kHz as calculated for the Finite-Element-models, i.e. 20 kHz. One should note that we recorded *p* ^sound^(*t*) not during the MRI-session but in a second measurement as described above. All IIR-filter were ensured to be causal and stable. Resulting filter coefficients are provided in Fleischer et al. (2022) for all models.

We computed the particle velocity $v_{g}^{\text{sound}}\left(t\right)$ at the glottis by deconvolution of *p* ^sound^(*t*) (multiplied with a Hann-window before processing) with the impulse response ${\hat{h}}_{g}^{l}$ of ${\hat{H}}_{g}^{l}$ using the python-code provided at http://www.rosettacode.org/wiki/Deconvolution/1D#Python.

Further, we derived the acoustic pressure $p_{g}^{\text{sound}}\left(t\right)$ at the glottis by filtering $v_{g}^{\text{sound}}\left(t\right)$ with ${\hat{H}}_{g}^{g}$ .

### 2.6 Electroglottographic spectrum and slope

We followed the guidelines given by Libeaux (2010) to estimate the spectral tilt or spectral slope of the experimentally determined EGG spectra. In short, we computed the short-time averaged wideband spectrum (STAWS) of the downsampled EGG-signal (sampling rate of 20 kHz) for each sample (bandwidth of 2 ⋅ *f*_*o*_, Hann-window with a sample size of 256, hop size of 128). According to Libeaux (2010), we fitted STAWS within the range between 2 ⋅ *f*_*o*_ and 10 ⋅ *f*_*o*_ with a linear polynomial regression in the log-log-scaled spectrum to compute the spectral slope. Finally, we derived the index of contacting *I*_*c*_ using FonaDyn (see Ternström (2019) for details).

### 2.7 Sound intensity

Finally, we evaluated the sound intensity $I_{g}^{\text{sound}}\left(t\right)=p_{g}^{\text{sound}}\left(t\right)\cdot v_{g}^{\text{sound}}\left(t\right)$ at the glottis. Consequently, for this study, $I_{g}^{\text{sound}}$ is determined by both numerically and experimentally derived data.

We filtered $I_{g}^{\text{sound}}\left(t\right)$ by a moving-average filter with a window length of 100 periods of the individual *f*_*o*_. We further computed the Hilbert transform $H\left\{I_{g}^{\text{sound}}\left(t\right)\right\}$ and derived the effective value $I_{g_{\text{eff}}}^{\text{sound}}$ of the magnitude of $H\left\{I_{g}^{\text{sound}}\left(t\right)\right\}$ . Multiplying of $I_{g_{\text{eff}}}^{\text{sound}}$ with the glottal area gives the sound power $P_{g_{\text{eff}}}^{\text{sound}}$ for each voice quality. Likewise, we computed $I_{g_{\text{eff}}}^{{\text{sound}}_{\text{norm}}}$ and $P_{g_{\text{eff}}}^{{\text{sound}}_{\text{norm}}}$ based on the to -23 LUFS normalized sound records *p* ^sound^(*t*) according to EBU-R128 using audacity (www.audacityteam.org). Theoretical insights of the concept of sound intensity can be found in Jacobsen (2014).

## 3 Results

### 3.1 Volumetric data of voice qualities and partial voluminas

**FIGURE 4:**
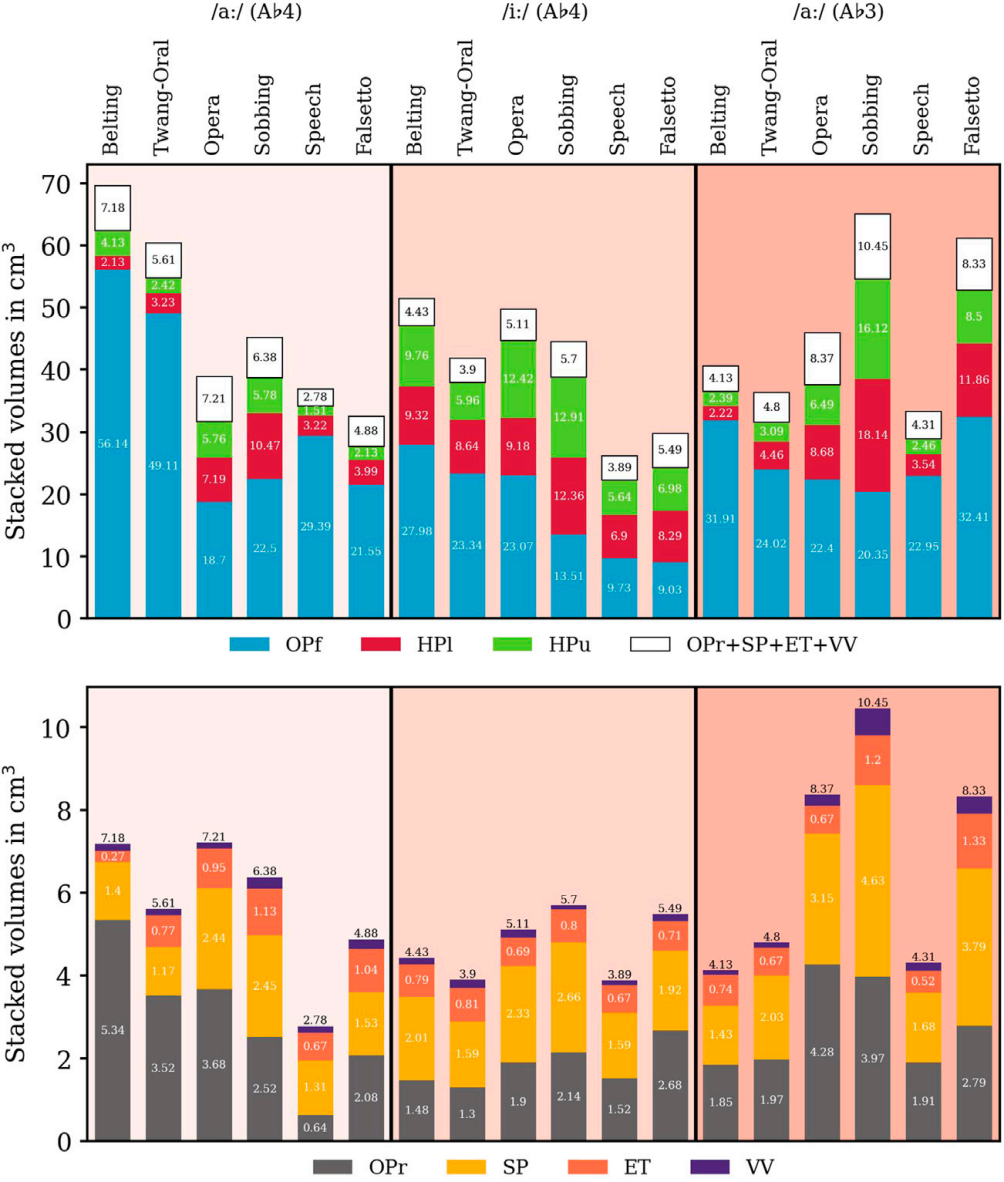
Volumes of sub-volumes for all voice qualities, vowels, and pitch values in cm^3^. Consistently to Figure 2, the oropharynx front (OPf) and the oropharynx rear (OPR) are symbolized by blue and gray color, and the upper hypopharynx (HPu) and the lower hypopharynx (HPl) are highlighted green and red, respectively. The epilarynx tube (ET), *Sinus piriformis* (SP), and ventricular volume (VV) are highlighted in orange, yellow, and purple, respectively. For clarity reasons, the anatomical order is not considered.

For comparison purposes and to evaluate their sizes, we determined the volumes of the vocal tract sub-structures as introduced in Figure 2. They depend on voice quality, vowel, and pitch (see Figure 4 and Table 1).

**TABLE 1:** Dimensionless OP/HP-ratio (Oropharynx/Hypopharynx) and vocal tract length L in cm, for all investigated voice qualities, vowels, and pitch values.

Vowel, pitch	Voice quality	OP/HP	L
/aː/(A♭4)	Belting	7.6	11.48
	Twang-Oral	6.8	12.21
	Opera	1.4	16.58
	Sobbing	1.2	15.31
	Speech	4.4	11.98
	Falsetto	2.6	12.68
/iː/(A♭4)	Belting	1.3	13.74
	Twang-Oral	1.4	13.41
	Opera	1	15.65
	Sobbing	0.5	14.8
	Speech	0.8	13.31
	Falsetto	0.6	13.96
/aː/(A♭3)	Belting	4.9	11.5
	Twang-Oral	2.5	12.68
	Opera	1.4	15.69
	Sobbing	0.6	16.7
	Speech	3	12.88
	Falsetto	1.4	14.14

While the length of the vocal tract differs by over 5.2 cm (Belting A♭4 /aː/: 11.48 cm; Sobbing A♭3 /aː/: 16.70 mm), the overall volume was more than doubled in the pool of voice qualities (Speech A♭3 /iː/: 26.33 cm^3^; Belting A♭4 /aː/: 69.73 cm^3^). We further found indications for a connection between vocal tract lengths and voice quality subgroups in the data. Opera and Sobbing models had the longest vocal tracts, Belting and Twang had the shortest, while Speech and Falsetto had a medium vocal-tract length. The length differences are equally pronounced for A♭4 /iː/ and /aː/ while they are less pronounced for A♭3 /aː/.

The differentiation of the subgroups was well represented in the analysis of sub-volumes, e.g., in the OP/HP ratio: megaphone configuration was characterized by OP/HP > 1, inverted-megaphone configuration by OP/HP < 1 and neutral configuration by OP/HP ≈ 1 for vowel /aː/, but not for vowel /iː/. The overall vocal tract volume differed widely between different models and no clear relation to voice quality subgroups became visible.

In intra-group comparison, the megaphone shape was more pronounced in Belting than in Twang. Here, for Belting, we observed larger oropharyngeal cavities, smaller hypopharynx, and smaller volumes of the epilarynx tube.

The inverted-megaphone shape was more emphasized in Sobbing compared to Opera. Here, the oropharyngeal volume was smaller, but hypopharyngeal subvolumes were larger. Falsetto models include a larger hypopharynx, whereas the oropharyngeal measures show no clear trend.

In all Belting vs. Twang, Opera vs. Sobbing, and Speech vs. Falsetto models, the lower hypopharyngeal volume is smaller in the first mentioned qualities. (the only exception is: Belting /iː/ compared to Twang /iː/ – here Belting was slightly larger than Twang).

### 3.2 Volume velocity transfer functions

**FIGURE 5:**
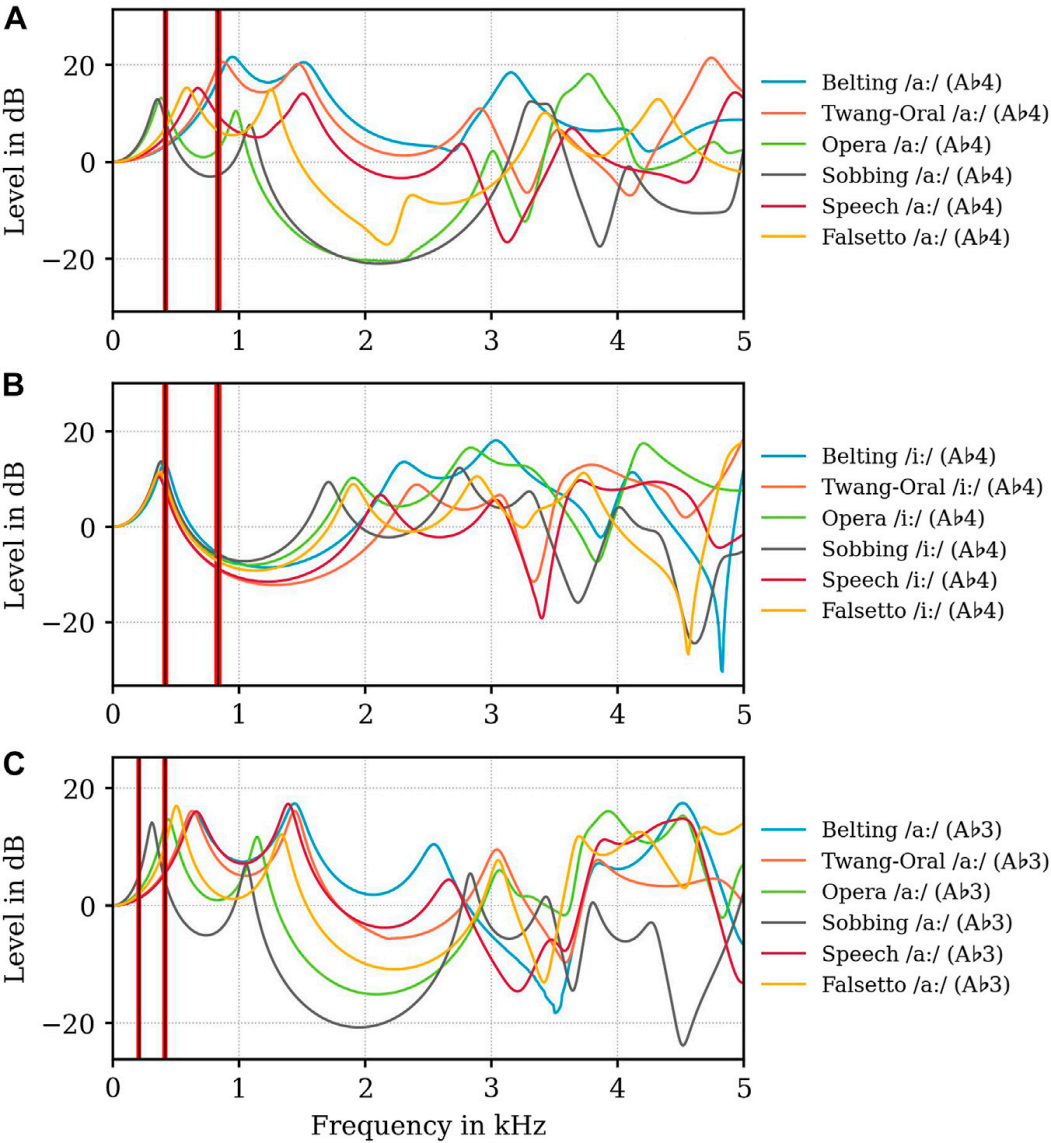
Level in dB of Volume velocity transfer functions $V_{g}^{l}$ for all voice qualities according to the legends in all sub-figures, for all vowels, and pitch values (**(A)** vowel /aː/ A♭4, **(B)** /iː/ A♭4, **(C)** /aː/ A♭3). Black vertical lines indicate the nominal fundamental frequencies *f*_*o*_ and the second harmonic 2 ⋅ *f*_*o*_, respectively. Red vertical lines indicate the range for the fundamental frequency values and the second harmonics audibly produced by the participant during MRI measurements. Because of the highly reproduced vocal exercise, red lines cannot be resolved in the figures and emerge a red area.

Volume velocity transfer functions $V_{g}^{l}\left(\omega \right)$ are displayed in Figure 5 in relation to nominal and measured *f*_*o*_ and 2*f*_*o*_ (nominal = vertical black lines and measured = vertical red lines). Maxima *f*_*Rn*_ and minima *L*_*Rn*_ are displayed in the Supplementary Data Sheet S1.

The level of $V_{g}^{l}$ show three different vowel strategies based on the position of *f*_*R*1_ and *f*_*R*2_ in the examined voice qualities that are most pronounced in A♭4 at 0.415 kHz (for /aː/) models (Figure 5A) in correspondence with the previously defined voice quality subgroups. Lowest frequency positions and amplitude levels are found in Opera and Sobbing models, medium in Speech and Falsetto models and highest in Twang and Belting models.

In Opera and Sobbing models *f*_*R*1_ is located in a close relation to *f*_*o*_ (*f*_*o*_ = 0.415 kHz, Opera: *f*_*R*1_ = 0.390 kHz, Sobbing: *f*_*R*1_ = 0.350 kHz) while in Belting and Twang models *f*_*R*1_ is located in close relation to 2*f*_*o*_ (2*f*_*o*_ = 0.830 kHz; Belting: *f*_*R*1_ = 0.950 kHz, Twang: *f*_*R*1_ = 0.870 kHz), while there is no relation between *f*_*R*1_ and/or *f*_*R*2_ and displayed harmonics for Speech and Falsetto (see Supplementary Data Sheet S1). Similar relation can be found in A♭3 /aː/ models, however less pronounced (Figure 5C). For A♭4 /iː/ models, (Figure 5B), different behavior can be noticed: *f*_*R*1_ is located here in close proximity (0.360–0.410 kHz) and close relation to *f*_*o*_ = 0.415 kHz, while for *f*_*R*2_ we found a similar distribution as for /aː/ (see Figure 5A).

Concerning economic considerations, resonance maxima in the area of 2–4 kHz are of specific interest as our auditory system is most sensitive here. For A♭4 (0.415 kHz), the highest resonance amplitude levels in this area occur in Belting and Opera vocal tracts: In Belting *f*_*R*3_ is located around 3 kHz for both /iː/ (3.040 kHz) and /aː/ (3.150 kHz). In Opera /aː/, a resonance maximum, which could be interpreted as *f*_*R*4_, is located at 3.770 kHz, while for /iː/, the highest level is found for *f*_*R*3_ at 2.840 kHz. For A♭3-models (0.208 kHz), only Opera has an eminent maximum in this frequency region at around 3.930 kHz. All other models show no specific resonance strategy or pronunciation for *f*_*R*3_ to *f*_*R*5_.

Worth mentioning are also pronounced resonance minima as these absorb energy and act as formant repellents (Dang and Honda, 1997; Delvaux and Howard, 2014). Distinct minima occur for all Sobbing models, one in the range between 3 and 4 kHz another in the range between 4 and 5 kHz. In Opera models the minima show relatively similar positioning in A♭4 /iː/ and A♭3 /aː/, but significantly less pronounced. For Speech and Twang very similar minima are found in all models between 3 and 3.5 kHz, while Falsetto models show very inconsistent behaviour here. Belting A♭4 /aː/ vocal tracts exhibit no pronounced minima, Belting A♭4 /iː/ has one briefly under 5 kHz and Belting A♭3 /aː/ has a minima at around 3.5 kHz.

The deviation between the first formant *F*1 derived from inverse filtering and *f*_*R*1_ calculated from $V_{g}^{l}\left(\omega \right)$ was between 4 and 260 Hz. The coefficient of determination *R* ^2^ of a linear regression of *F*1 and *f*_*R*1_ was 0.68 (see Supplementary Data Sheet S1).

### 3.3 Filter approximation

**FIGURE 6:**
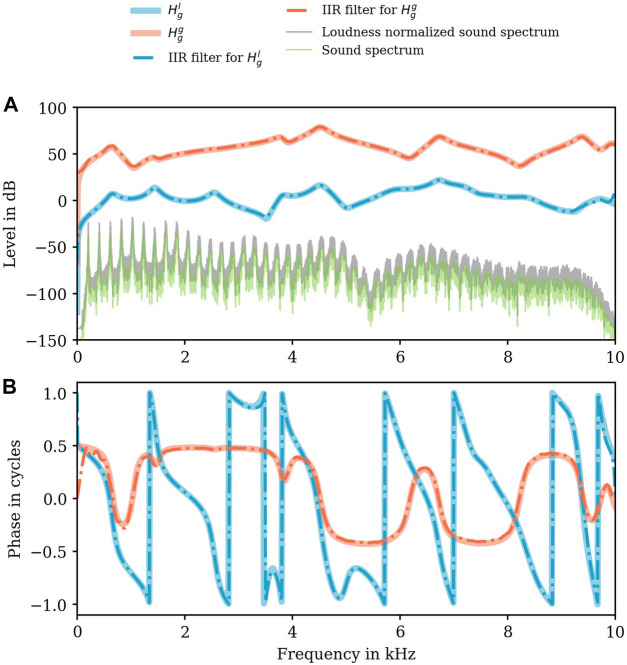
Level in dB **(A)** and phase responses **(B)** of the numerically determined transfer characteristics $H_{g}^{l}$ (light blue, thick lines), $H_{g}^{g}$ (light orange, thick lines), and their IIR-approximates (chain lines) according to the legend exemplarily shown for Belting /aː/ (A♭3). Additionally, the frequency spectrum of the original (green color) and loudness normalized sound data (gray color) are plotted in the upper panel **(A)**.

The numerically determined resonance frequencies of the ratio $H_{g}^{l}$ (Figure 6A) match those of the $V_{g}^{l}$ . Nevertheless, as reasoned by the different lip area and glottal area for each model, this measure is not normalized to 0 dB as in the case of the $V_{g}^{l}$ (see Figure 5). In contrast, the resonance frequencies of $H_{g}^{g}$ differs only slightly from $H_{g}^{l}$ . Further, their overall shape in the frequency domain and their level differs significantly. For both $H_{g}^{l}$ and $H_{g}^{g}$ , the IIR approximation was in great accordance with the target.

The RMS error from $H_{g}^{l}$ was not greater than 8.1 Pa⋅s/m. For $H_{g}^{g}$ , the RMS error of the IIR-approximant was not greater than 417.06 Pa/Pa (see Figure 6A). Exemplarily shown in Figure 6B, we always observed a low phase difference between $H_{g}^{l}$ and its IIR-approximant and $H_{g}^{g}$ and its IIR-approximant.

Comparing the envelopes of the sound spectra (raw data and loudness normalized sound spectrum) with $H_{g}^{l}$ , these envelopes do not contain the spectral features of $H_{g}^{l}$ . Here, exemplarily shown for Belting in A♭3 (/aː/), *f*_*R*2_ can be estimated in the sound envelope, but *f*_*R*1_ and *f*_*R*3_ not (Figure 6A).

### 3.4 Acoustic and electroglottography related measures

**FIGURE 7:**
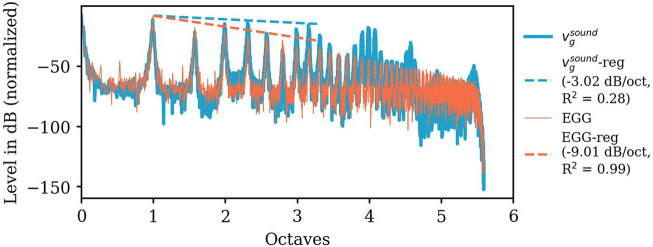
Amplitude spectra (in dB, normalized to its maximum) of the particle velocity (computed by a post-processing step combining experimentally and numerically derived data) at the glottis $v_{g}^{\mathrm{sound}}$ (blue color), the experimentally determined EGG-spectrum (orange color), and their spectral slopes based on short-time averaged wideband spectra exemplarily shown for Belting /aː/ (A♭3). The abscissa shows the log_2_-transformed and the normalized frequency (normalized to the individual fundamental frequency *f*_*o*_) in octaves (zero corresponds to the fundamental frequency and the first octave to the first harmonics, respectively).

**FIGURE 8:**
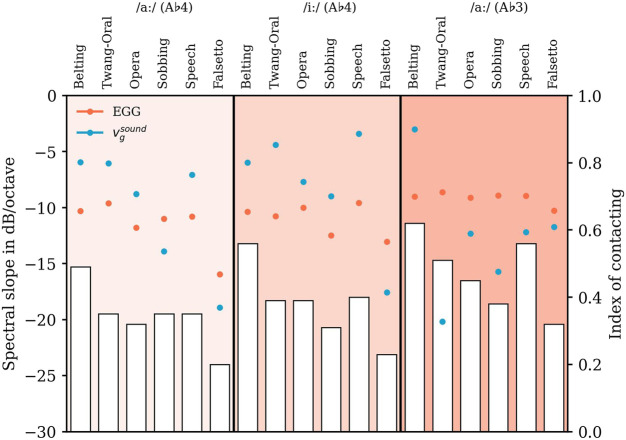
Slopes in dB/octave (left ordinate) of the experimentally derived EGG-spectra (circles, filled in orange) and the computed particle velocity $v_{g}^{\mathrm{sound}}$ at the glottis (circles, filled in blue). These quantities are shown for all voice qualities, vowels, and pitch values (see abscissa). Additionally, the Index of contacting *I*_*c*_, limited by zero and one (corresponding to the right ordinate), is highlighted with white bars. The results for two vowels (/aː/ and /iː/) and the pitch values are filled separately with different shades of orange.

EGG spectral tilt and calculated particle velocity at the glottis $\left(v_{g}^{\mathrm{sound}}\right)$ are exemplary demonstrated in Figure 7 for Belting /aː/ (A♭3). Figure 8 displays all slopes of the EGG-spectra and $v_{g}^{\mathrm{sound}}$ as well as the EGG derived measure *I*_*c*_. *I*_*c*_ ranges between 0.2 and 0.62 (see Supplementary Data Sheet S1). While some subgroup pairs differ significantly in their *I*_*c*_-values (Belting/Twang and Speech/Falsetto), others show almost the same value for all vowel and pitch combinations (Opera/Sobbing). Falsetto, which is indented to be produced with “stiff” (abducted) vocal folds, shows the lowest *I*_*c*_ and Belting, which should be produced with “thick” vocal folds and high larynx according Estill Voice Training^®^ (Steinhauer et al., 2017), shows the highest *I*_*c*_. The *I*_*c*_ of Speech, was increased in A♭3, while for A♭4 the level of *I*_*c*_ was comparable to the voice qualities with “thin” vocal folds. Overall, the spectral slope values of $v_{g}^{\mathrm{sound}}$ for A♭3 are lower than for A♭4: Whereas for A♭3, *I*_*c*_ tends to have higher values ranging from 0.32 to 0.62, for A♭4, we found *I*_*c*_ to be 0.2 to 0.56 (see Supplementary Data Sheet S1). Further, a vowel-related effect seems minor.

Overall, the range of variation of the EGG slope is much smaller compared to the $v_{g}^{\mathrm{sound}}$ slope. For EGG all values lie between -8.61 and -15.94 dB/octave (Figure 8; Supplementary Data Sheet S1). Slight downward outliers can be seen for all Falsetto as well as Sobbing phonation at A♭4 /iː/. In comparison, the calculated $v_{g}^{\mathrm{sound}}$ slopes show a significantly greater variability with values between -3.02 and -20.2 dB/octave (see Supplementary Data Sheet S1). Again highest values occur for Belting and lowest for Falsetto for all A♭4 models, while for A♭3 lowest value was found for Twang.

### 3.5 Calculated sound intensity

**FIGURE 9:**
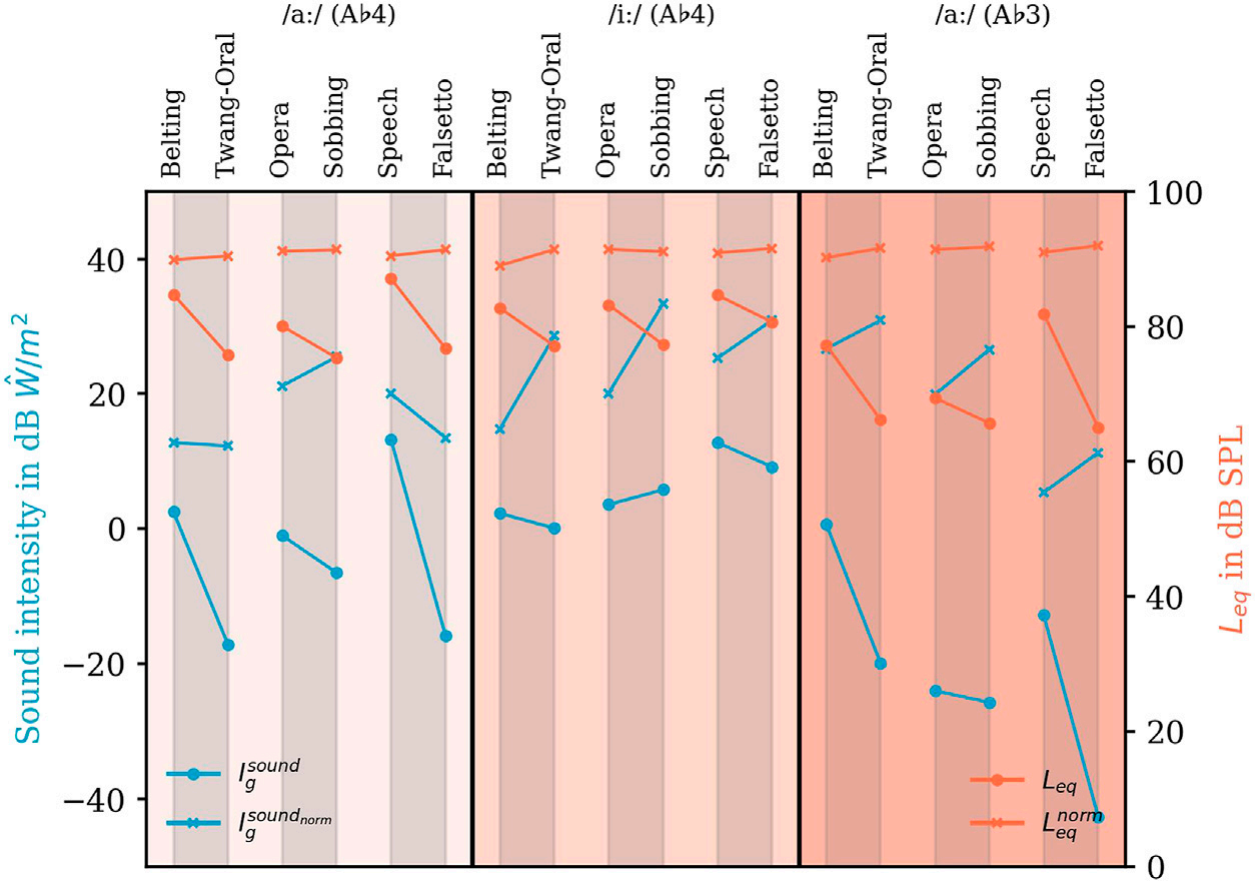
Sound intensity $I_{g}^{\text{sound}}$ (in dB $\hat{W}$ /m^3^, left ordinate, blue color) and equivalent sound pressure level *L*_*eq*_ (in dB SPL, right ordinate, orange color), and their loudness-normalized counterpart ( $I_{g}^{{\text{sound}}_{\text{norm}}}$ and $L_{eq}^{\mathrm{norm}}$ ) for all voice qualities, vowels, and pitch values according to the abscissa. The results for two vowels (/aː/ and /iː/) and the pitch values are filled separately with different shades of orange. The pairwise combination of two results each is denoted with gray colored area.

Calculated sound intensity for original $\left(I_{g}^{\mathrm{sound}}\right)$ and normalized $\left(I_{g}^{sound_{\mathrm{norm}}}\right)$ sound data is displayed in blue in Figure 9 in relation to the respective *L*_*eq*_ in orange.

To highlight the differences in the previously defined resonance subgroups, we connected these subgroups by lines. Additionally calculated values for sound power ( $P_{g}^{\mathrm{sound}}$ and $P_{g}^{sound_{\mathrm{norm}}}$ ) are displayed in the Supplementary Data Sheet S1 to check for the influence of the glottal area on the values. Here, we found a high congruence between power and intensity values. Sound intensity calculated from loudness normalized sound data (blue crosses) represents the amount of acoustic power necessary to excite the respective vocal tract. It was higher in Sobbing, Twang, and Falsetto. These voice qualities required more sound intensity to achieve the same loudness as Opera, Belting, and Speech (with an exception for Belting vs. Twang A♭4 /aː/ that were similar, and Speech vs. Falsetto A♭4 /aː/ where Speech needs a higher sound intensity).

The values calculated from original sound data display the sound intensity used to reach the respective loudness (see round marks in Figure 9). Additionally, the equivalent sound pressure level *L*_*eq*_ (calculated for the whole analyzed sound sample) according to ISO 3740:2019 is displayed as a plausibility check since the audio data and the calculated intensity data originate from two different measurement times. Here, except for the pair Opera -Sobbing A♭4 /iː/, all models show a higher intensity for higher *L*_*eq*_ which also seems to correlate in magnitude.

As expected, the loudness normalized data show a very homogeneous level (orange crosses in Figure 9). The non-normalized data from each examined group show a louder (Opera/Belting/Speech) and a softer (Sobbing/Twang/Falsetto) voice quality.

Generally, *L*_*eq*_ was lower for A♭3 compared to the respective voice quality for A♭4. To reach the higher *L*_*eq*_ for vowel /aː/ phonation highest sound intensity was necessary for Belting, lower for Opera, and low for Speech, while for /iː/, this difference was minor.

### 3.6 Sound intensity in relation to geometric data

In a final step, the calculated intensity values were considered in relation to the vocal tract configuration. Subvolumina and intensity are summarized in the Supplementary Data Sheet S1 but due to the very small data pool, statistical correlations were not conducted. Nevertheless, a simple visual inspection reveals that there is no clear relationship between the magnitude of the intensity values and the volumes of most of the different parts of the vocal tract. One exception here is the lower hypopharyngeal volume: Here, the arrangement of the values in the plot might suggest a correlation: The smaller the (lower) hypopharyngeal volume, the higher the calculated intensity value of the voice quality. Additionally, this is reflected by a higher OP/HP-ratio associated with higher sound intensity.

## 4 Discussion

### 4.1 Preliminary remarks about the definition of voice efficiency

The presented study aims to increase the knowledge of resonance strategy and related voice efficiency for various voice qualities.

“Voice efficiency” can be viewed and analyzed from various points of view. In our context, we define the phrase “efficient voice production” as a low sound intensity at the glottal level required to acoustically excite the respective air column in the space between vocal folds and lips. Here, the lower the sound intensity needed for a defined sound pressure level, the higher the degree of efficiency. Additionally, we will discuss some further aspects of the perceived loudness.

In addition to these economic considerations, the examined types of voice production also have a purely artistic justification since they are linked, for example, with certain emotions regarding the performer in the listener’s perception. Thus, our study does not aim to suggest one or another voice quality but to increase awareness of the underlying principles, especially when the vocal demand is high.

### 4.2 Volume velocity transfer functions and vocal tract configuration

The investigated six voice qualities can be assigned to three different subgroups according to their vocal tract configuration (Titze and Worley, 2009; Titze et al., 2011, 2017). Titze et al. (2011) based their assumptions on predicted or assumed vocal tract area functions for famous singers of different genres based on their mouth configurations, corresponding frequency spectra, and calculated inertagrams. To the best of our knowledge, this is the first study to imagine these configurations with different voice qualities in one professionally trained singer.

The results of our study support Titze’s assumptions concerning the configuration of the vocal tract (see partial volume distribution) and related position of the lowest two resonances. But, the differences are generally more pronounced for /aː/ compared to /iː/ and for A♭4 compared to A♭3 models, which could be related to the substantial difference in tongue position. Volumetric and calculated acoustic data is thus generally discussed in the context of these three subgroups.

#### 4.2.1 Group 1: “Inverted-megaphone” configuration’ models Opera and Sobbing

The inverted-megaphone configuration of the vowel tract is found typically in western-style classically trained singers (Titze and Worley, 2009; Titze et al., 2011) (except for high soprano singing (Köberlein et al., 2021; Mainka et al., 2021)), and corresponds to Sobbing and Opera in the qualities we have studied. In our data, these models show the lowest resonance frequencies *f*_*R*1_ and *f*_*R*2_.

The lowering of *f*_*R*1_ and *f*_*R*2_ might be beneficial to avoid instabilities that might occur when crossing resonances with 2 ⋅ *f*_*o*_ or *f*_*o*_ when *f*_*o*_ rises (Titze and Worley, 2009). In both A♭4-models, *f*_*R*1_ is positioned close below *f*_*o*_. Whether this association occurs by chance or represents the onset of tuning for higher phonation cannot be conclusively answered from our data. A consistently lower formant *F*_1_ for classical singing compared to Belting was also reported by Sundberg et al. (2012) in different vowels in acoustic analysis of a single subject.

Regarding economic considerations of higher vocal tract resonances, Opera and Sobbing showed very different strategies. For Sobbing, we found distinctive antiresonances, but for Opera, we found more pronounced resonance maxima. Enhancement of spectral energy in the area of 2–4 kHz is a well-known phenomenon of the western classical singing strategy called singers’ format and was assigned to a direct supraglottic constriction in relation to the widening of the hypopharynx further cranially (Sundberg, 1974).

In previous studies, an increased constriction of the epilarynx tube was associated with an increase in radiated sound pressure level in classically trained male singers (Mainka et al., 2021). However, the reduction of the lower hypopharyngeal volume from Sobbing to Opera seems to be even more pronounced than the difference in the Epilarynx tube volume. A simple magnitude relationship between these two regions as the cause of the distinctive resonance change seems unlikely.

Two side cavities, the vallecula and the *piriform sinus* connect to the lower hypopharynx.

The *piriform sinus* introduces an anti-resonance around 4–5 kHz (Dang and Honda, 1997; Honda et al., 2010; Delvaux and Howard, 2014). Delvaux and Howard (2014) postulated that these side cavities act as “formant repellent” pushing *f*_*R*1_ to *f*_*R*4_ lower (Delvaux and Howard, 2014). Dang and Honda (1997) postulated that these antiresonances tune the vocal tract transfer function to make the singers’ formant more prominent. Additionally, Dang and Honda (1997) found only minor differences in the frequencies of the antiresonances for different vowels in speech and singing. Reasons are small changes in the geometry of the *piriform fossae* from speech to singing.

Indeed, that is consistent with our data, where the size differences of the piriform sinus between Opera and Sobbing were minor (e.g., A♭4 /aː/), while the expression of antiresonances shows major differences in the same models.

The vallecula, in turn, was discussed to have a similar influence on the transfer function (but at lower frequencies around 4 kHz) (Vampola et al., 2015; Feng and Howard, 2021). In our data, we included the volume of the vallecula in the hypopharyngeal region which was larger in Sobbing compared to Opera independent of vowel and pitch. The assumption of Feng and Howard (2021), that a large vallecula is detrimental to the singing formant, is supported by our data.

In contrast to previous assumptions that a wide *sinus morgagni* is beneficial for the singers’ formant cluster Sundberg (1974), our data show a heterogeneous behavior of the ventricular volume for Opera compared to Sobbing, which, e.g., for A♭4 /aː/ is twice as large in Sobbing and in A♭4 /iː/ the other way around. The influence on the singing formant thus seems minor.

In summary, we found the large side cavities in Sobbing tend to have a suppressing effect because these cavities reduce the amplification and, thus, the power transfer at frequencies important for the perceived loudness. From a pedagogic perspective, this result is interesting as in western classical voice pedagogy, the widening of the pharynx is often thought to be associated with an increase in voice intensity (Faulstich (2020), p. 50). However, our data indicate a decrease in voice efficiency when the widening of the pharynx is associated with increasing volumes of side cavities.

#### 4.2.2 Group 2: “Megaphone” models Belting and Twang

Megaphone-shaped vocal tract models are reported to be typical in musical theatre (Echternach et al. (2014a)) and “twang-like singing” (Saldias et al. (2019)) as well as very high-pitched Soprano singing (Köberlein et al., 2021). In our dataset, they are found for Belting and Twang as defined by Estill Voice Training^®^. We found high resonance frequencies *f*_*R*1/2_ for both models. That is in conjunction with a light vowel color and an open vowel configuration mentioned by Titze et al. (2017).

According to the literature for /aː/, a close relation is found of the first resonance frequency *f*_*R*1_ that is placed slightly above 2*f*_*o*_ for A♭4 (Titze and Worley, 2009; Bourne and Garnier, 2012; Sundberg et al., 2012). That is certainly efficient for voice production since the level of 2*f*_*o*_ (which is supposed to be strong in the spectrum of Belting (Stone et al. (2003)) is amplified even further.

Bourne and Garnier (2012) described a 2*f*_*o*_−*f*_*R*1_ tuning up to 700 Hz (pitch F5) for Belting, whereas Köberlein et al. (2021) detected a value of about 1.4 kHz (pitch F6) for *f*_*o*_−*f*_*R*1_ in very high western classically trained soprano singing, respectively, for the same rationale. These findings indicate a possible natural upper end of the further elevation of *f*_*R*1_. That supports the idea that Belting performs stable in a limited pitch ranges or that a change of strategy is required for higher belting. It is supposed that avoiding the crossing of 2*f*_*o*_ and *f*_*R*1_ is beneficial to prevent instabilities due to source-filter interactions (Maxfield et al., 2017). For A♭3, we observed *f*_*R*1_ to be significantly higher than 2*f*_*o*_, which is in unison with Titzes suggestion that belting is associated with *f*_*R*1_ > 2*f*_*o*_ and not the definition of Bourne and Garnier (2012) who suggested a *f*_*R*1_ = 2*f*_*o*_ tuning.

Interestingly, we did not observe this typical 2*f*_*o*_−*f*_*R*1_ tuning behavior for the vowel /iː/. Here, only *f*_*R*2_ was higher than in the other groups, while *f*_*R*1_ amplified the level at the *f*_*o*_. That might also be an advantage from an economic point of view.

In summary, our data confirm the relationship of 2*f*_*o*_−*f*_*R*1_ only for A♭4, however, *f*_*R*1_ (respective *f*_*R*2_ for /iː/) is always most elevated compared to the other model groups and positioned higher then 2*f*_*o*_.

Concerning the behavior of higher resonances, it is interesting to note that the relationship between Belting and Twang is similar to that between Opera and Sobbing. Here, Twang exhibits significantly stronger antiresonances in the transfer function in this region than Belting. That finding is more pronounced for A♭4. High levels at the resonance maxima in Belting, which certainly have a positive effect on sound pressure level and thus the efficiency, are weakened in amplitude and shifted by antiresonances for Twang. This links to anatomical measures, namely the hypopharyngeal volumes. Twang shows a larger HPl than Belting for vowel /aː/. More precisely, reducing the volume of the vallecula might be a crucial factor for increased economic usage of the vocal tract. It may further result in an increased sound pressure level caused by the weakened effect of levels at antiresonance frequencies.

Notably, this outcome may be vowel-dependent. For /iː/, the vallecula is not shaping a lateral cavity but is part of the hypopharynx. For /aː/, the retraction of the tongue and thus the epiglottis truncates this space to a greater extent.

#### 4.2.3 Group 3: “Neutral” models Speech and Falsetto

Neutral models show a middle position of the lowest two resonance frequencies with no direct association with any partials of the spectrum.

In our data, *f*_*R*1/2_ for Speech was slightly higher than for Falsetto, which could also be associated with a slight reduction of the vowel tract length. We found a shift to higher values for most of the higher resonance frequencies, but both models do not show pronounced resonance frequencies. Further, *f*_*R*3−5_ are spanning a large range in the frequency spectrum.

### 4.3 EGG related data on vocal fold vibration and spectral slope

We analyzed EGG data to detect the relationship between spectral properties of the acoustic outcome and vocal fold vibration characteristics. More precisely, we analyzed the index of contacting *I*_*c*_ as proposed by Ternström (2019). As a heuristic metric, *I*_*c*_ for strong phonation is in the order of one and tends to zero for soft phonation or no collision.

Considering Figure 8, our data show a tendency of *I*_*c*_ to be decreased going from A♭3 to A♭4. That finding is supported by the literature on female voices, where a decrease in vocal fold contact quotient–not identical to *I*_*c*_–with increasing *f*_*o*_ was already described (Traser et al., 2021a) as well as an increase of the open quotient with *f*_*o*_ based on high speed imaging data Echternach et al. (2017). On the other hand, the *f*_*o*_ dependency of *I*_*c*_ could also be a result of more harmonics dropping out of the 10 kHz analysis band that became more prominent at higher *f*_*o*_ (L˜a and Ternström, 2020). The latter seems more plausible because the volume did not substantially change between voice qualities and pitches (see Figure 9).

To further distinguish vibratory states of the vocal folds, we analyzed the EGG-spectrum and its spectral slope or decay, respectively (Selamtzis and Ternström, 2014). The EGG waveform may be a good approximation to the change in medial contact area between the vocal folds during phonation with limitation in the contacting phase according to Hampala et al. (2016). The greater the vocal fold contact area, the lower the spectral slope (Enflo et al., 2016) and the higher the vocal intensity (Sapienza et al., 1998). To relate the EGG data directly to the glottal flow data, we computed the particle velocity $v_{g}^{\mathrm{sound}}$ at the glottis by deconvolution of the audio recordings using IIR-filter. To date, $v_{g}^{\mathrm{sound}}$ cannot be measured directly and is always an estimation which is also the case for this study. Comparing the EGG spectral slope with the slope of $v_{g}^{\mathrm{sound}}$ , we found no general concordance of these measures. While the EGG spectral slope varied less for all trials, the spectral slope of $v_{g}^{\mathrm{sound}}$ showed large deviations triggered by the spectral properties of the vocal tract transfer functions. Nevertheless, we found correlations between the intended and calculated voice source and the EGG data.

For Falsetto, we found the largest decay of spectral slope in EGG-data and the lowest *I*_*c*_ in the dataset (especially for A♭4), indicating a vibration pattern with marginal vocal fold closure and a voice source with a low energy level of all harmonics. The latter is reflected evidently by a great decay in the spectral slopes of $v_{g}^{\mathrm{sound}}$ .

For Belting, we found the lowest decay of the spectral slope and the highest *I*_*c*_, indicating a vibration pattern with high vocal fold contact and a voice source with high energy in higher harmonics. This is in accordance with Stone et al. (2003) who also describe a higher EGG closed quotients for Broadway compared to operatic singing.

Compared to Belting, Twang significantly drops in *I*_*c*_, but the spectral slope of the EGG-data is very similar for all pitches and vowels. Here, the spectral energy of $v_{g}^{\mathrm{sound}}$ is larger than in other as “thin” intended vibration patterns. That could be associated with the elevation and related compression of the larynx which would increase vocal fold adduction.

Speech was characterized by a low decrease in spectral energy, comparable to Belting for A♭4 but lower for A♭3 with *I*_*c*_ nevertheless more in line with that of Twang and Opera even if “thick” vocal fold is intended.

Comparing Opera and Sobbing, we found only a slight decrease in *I*_*c*_ for Sobbing (similar values for A♭4 /aː/). Also, the slope of $v_{g}^{\mathrm{sound}}$ was slightly higher for opera. However, it indicates a similar oscillatory behavior combined with a voice source with a comparatively medium decrease of energy in the spectrum.

Considering that Opera, Belting, and Speech each achieved comparably high *L*_*eq*_, and Sobbing, Twang, and Falsetto similarly low *L*_*eq*_, one could speculate a difference in the dependence of the *L*_*eq*_ on the voice source (and the vocal fold vibration) and the resonance properties. The *I*_*c*_ decreases strongly from Speech to Falsetto with large difference in the slope of $v_{g}^{\mathrm{sound}}$ . However, these two voice qualities are similar in their resonances by not applying an explicit amplification strategy as decribed above.

In contrast, Opera and Sobbing differ rather less in their vocal fold vibration and voice source but are more pronounced in the resonance properties for higher frequencies.

Moreover, Belting shows a higher vocal fold contact and distinctive resonance maxima for higher frequencies than Twang. However, the decay of the spectral energy of the calculated voice source $v_{g}^{\mathrm{sound}}$ is similar.

However, dependent on the chosen *f*_*o*_ and its relation to the resonance frequencies, the EGG-signal is slightly affected by the vocal tract geometry and its reactance by Level 2 interaction (pitch shifts or instabilities in *f*_*o*_) (Maxfield et al., 2017) and does not involve glottal flow directly (Palaparthi et al., 2017). However, caused by Level 1 interaction, the spectral properties of glottal flow are affected by the individual shape of the vocal tract to a greater extent (Maxfield et al., 2017). More precisely, Level 2 interaction seems not to re-shape the spectral tilt of the EGG-signal, whereas Level 1 does.

### 4.4 Calculated sound intensity as a measure for vocal strength in relation to volumetric data

In the previous sections, we discussed the voice efficiency based on sound pressure level (expression of resonance maxima in the range of 2–4 kHz of volume velocity transfer function). In addition, we used acoustic recordings, filtered with the known acoustic properties, to analyze the voice source information contained therein and considered together with the EGG-based information on vocal fold vibration.

In a linear system, the separate investigation of voice source and acoustic properties would be sufficient for discussing voice efficiency. However, evidence for non-linear source-filter relationships as a relevant factor for human voice production is strong (but still not sufficiently understood). For general description of non-linear interaction see e.g. Titze (2008), for more recent *in vivo* analysis of professional voices see Echternach et al. (2021). However, there is consent that vocal tract configuration impacts voice source energy transfer. Thereby the acoustic pressure in the vocal tract can strengthen or disturb vocal fold vibration itself (Level 2 interaction (Maxfield et al., 2017)) or only influence the harmonic content (Level 1 interactions).

The presented investigation aims to propose a new measure to quantify the effort necessary for a voice source to excite a specific vocal tract.

In the chosen approach, we calculated the averaged particle velocity and the acoustic pressure at the glottis (see method section). One should note that the derived quantity “sound intensity” $I_{g}^{\mathrm{sound}}$ is not identical to *L*_*eq*_ derived in front of the open mouth. Considering units of *W*/*m* ^2^ of $I_{g}^{\mathrm{sound}}$ , this quantity represents acoustic power, normalized to the area of the source, radiated from a source. Further, we derived this quantity at the input of the vocal tract, not in front of the mouth. Generally speaking, the easier an air column within a vocal tract is to excite, the lower the sound intensity needed to achieve *L*_*eq*_. An increase in $I_{g}^{\mathrm{sound}}$ is thus related to a less economic acoustic energy transfer from the voice source through the vocal tract. A high glottal intensity in this context would imply that the vocal tract configuration is less economical for energy transfer and a higher “vocal strength” is needed.

In the intra-group comparison of different voice qualities investigated in this study, we observed less glottal intensity for Belting vs. Twang, Speech vs. Falsetto, and Opera vs. Sobbing to reach the same *L*_*eq*_ (except for Belting-Twang A♭4, which had almost identical values). Belting, Speech, and Opera seem more efficient than Twang, Falsetto, and Sobbing. This result is also widely consistent with data by Estill et al. (1990), who discussed voice efficiency of the same voice qualities but defined it as the ratio of sound pressure level and airflow at the lips in a single-subject experiment. The authors included Twang generally among the efficient qualities but showing also a reduction of efficiency for lower and medium pitches, respectively, comparable to our data. In that study, the efficiency of Twang was similar to that of Belting for *f*_*o*_ around 600 Hz in terms of efficiency, a value for *f*_*o*_ that we did not investigate in our study. Of course, the economy measure used by Estill et al. (1990) considers a different aspect of voice efficiency as measured in our study.

For the vowel /iː/, $I_{g}^{\mathrm{sound}}$ were somewhat higher (more acoustic power needed) than most of the voice-quality-matching vowel tract at /aː/.

Because of the novelty of this measure in the context of voice production, we found, to our best knowledge, no comparative values in the literature. But, our proposed tendencies of $I_{g}^{\mathrm{sound}}$ regarding efficient energy transfer may also be reflected by the vocal tract reactance, estimated by considering the energy transfer of–with the vocal tract–interacting voice sources. Based on non-linear source-filter-interaction, a positive vocal tract reactance (inertance) may delay the glottal flow pulse, thereby increasing the maximum flow declination rate and the energy forwarding into the vocal tract and the other way around for negative reactance. If located in this positive reactance (inertance) frequency range, an enhancement in the amplitude of the partials of the glottal flow may be achieved (Titze, 2008; Titze et al., 2021). Thus, the inertance may help to quantify the degree to which the airway assists the source in vibration and enables energy transfer at the respective frequency. As it is a frequency-dependent measure, it is often displayed according (Titze, 2020; Titze, 2006; Titze and Worley, 2009; Titze et al., 2011) as an inertagram (former inertogram). According to this concept, one would expect a sharp narrow peak with high inertance prior to a vocal tract resonance.

Considering our data, in Belting and Twang A♭4 /aː/, we found 2*f*_*o*_ located just below the peak of *f*_*R*1_. That means the second harmonic would most likely match the maximum of the inertance, further strengthening the voice source and thus facilitating energy transfer. Congruently, both vocal tracts have lower sound intensity values compared to, e.g., A♭3 /aː/, where 2*f*_*o*_ is far lower than the peak of *f*_*R*1_.

An important factor for sound power transfer and source filter interactions might also be an epilaryngeal narrowing. Titze et al. (2021) found that this narrowing raises the inertance of the whole vocal tract air column.

Also, the glottal resistance (and thus vocal fold vibration) plays an important role. According to the maximum power transfer theorem, a wide ET would require a low glottal resistance for maximum power transfer. Conversely, a narrow ET requires a high glottal resistance (more adduction) for maximum power transfer (Titze, 2002). This was confirmed experimentally by Döllinger et al. (2006), who found a decrease in phonation threshold pressure through impedance matching of the glottal source and the vocal tract. That would suggest the optimal efficiency relates to the configuration of the lower vocal tract and hence the individual voice quality.

In our data, we found the smallest ET volume of 265.24 mm^3^ for Belting (A♭4), while we found a significant increase to 740.47 mm^3^ of this sub-volume for Belting A♭3 /aː/. Both show a high *I*_*c*_ of 0.49 and 0.62, respectively, which presumably would be associated with an increased glottal resistance. Interestingly, $I_{g}^{sound_{\mathrm{norm}}}$ is significantly higher for A♭3 /aː/ (26.7 dB resp. W/m^2^) than for A♭4 /aː/ (12.7 dB resp. W/m^2^). That implies that for A♭3 /aː/, more acoustic power is needed to reach the same normalized *L*_*eq*_, and thus be considered less economical than Belting A♭4 /aː/. In line with this, Twang A♭4 /aː/, for example, exhibited a lower *I*_*c*_ of 0.35 compared to Belting and a larger ET volume of 765.12 mm^3^ with $I_{g}^{sound_{\mathrm{norm}}}$ of 12.4 dB resp. W/m^2^ an equally value as determined for Belting A♭4 /aː/. For A♭4, also Sobbing models featured less favorable power transfer and sound intensity, identical to Opera, which was again associated with smaller ET volume.

Due to the single-subject design, we did not perform correlation analyses on the vocal tract configuration as represented by the partial volumes with the calculated sound intensities. For those interested in details, we attached plots of sub-volume data and sound intensity values for visual inspection analysis in the Supplementary Data Sheet S1.

Despite these examples, we found that cross-subgroups, the sole volume of the ET seems to have no relation to the sound intensity needed to excite the vocal tract. That would be congruent with the maximum power transfer theorem that implies that the ET narrowing should be chosen according to the respective voice source and should not be a general recommendation. Additionally, we observed no clear cross-subgroup relationship between sound intensity and any other sub-volume parameter. That underlines that the reduction for single morphometric parameters seems not to be valid within the data of this study for evaluating voice efficiency. More precisely, one should incorporate the morphometric complexity of the whole vocal tract and the interaction between sub-volumes (see Arnela and Ure˜na (2022)) when the power transfer from the glottis to and through the vocal tract is analyzed.

A decrease of HPl (in percent of the whole vocal tract volume) might relate to an increase in sound intensity (see Supplementary Data Sheet S1). However, all Belting and Twang /aː/ phonations were outliers suggesting different dimension-relation for megaphone models. Additionally all /iː/-models showed higher sound intensity values compared to respective /aː/ models suggesting, more effective power transfer for vowel /aː/ (with general decrease in hypopharyngeal volume).

To what extent the suggested value of the sound intensity correlates with muscular tension or the necessary phonation threshold pressure remains unclear. Also, it is not yet possible to assess the extent to which sound intensity is related to mechanical stress on the vocal folds. Moreover, regarding “sound intensity”, it is interesting to note that the acoustic power emitted and radiated by a sound source contains only a tiny portion of the energy conversion of almost any source (Jacobsen, 2014).

### 4.5 General discussion of data plausibility and limitations

The Finite-Element-Method for solving the scalar Helmholtz-equation for the acoustic pressure as the unknown quantity is an appropriate procedure proven by direct comparison with experiments for three-dimensional vocal tract replicas (Birkholz et al., 2020). Nevertheless, specification of adequate boundary conditions at the vocal tract wall might be a crucial factor for correct mimicking of different vowels and voice qualities (Fleischer et al., 2015). However, the application of a constant, frequency and spatially independent wall impedance, as we did for the present study, cannot capture the spectral properties of the realistic transfer function in detail. To minimize the unknowns in the whole dataset, we believe that this limitation does not bias our results too much (see Švancara and Horáček (2006); Arnela et al. (2013); Zappi et al. (2016); Arnela and Ure˜na (2022) for further insights).

Based on the numerically determined transfer functions, we calculated IIR-filter approximations for all models. As demonstrated, the frequency characteristic of these filters does not only capture the FE results in amplitude but also in phase. Therefore, no significant time delay is caused just by the filter. Unfortunately, finding an appropriate set of parameters for the poles and zeros was done by hand. However, careful visual checking of all IIR approximations confident us that the presented method is valid for subsequent deconvolution of the audio signals of each task.

It is known that the vocal tract morphology depends on *f*_*o*_ (Köberlein et al., 2021; Echternach et al., 2014b). One should note that this adaption process should not confound with the non-linear interaction of the filter (vocal tract) and the source. More precisely, two interaction levels are known. Either it reflects changes in the spectral distribution of the glottal flow (Level 1 interaction) or changes in *f*_*o*_ caused by the specific reactance of the vocal tract (Level 2 interaction) (Maxfield et al., 2017). To our best knowledge, a reverse interaction, meaning changes in the vocal tract morphometry caused by the source signal, is not to be expected. That led us to assume that the deconvolved audio signals, based on our presented transfer function and derived IIR approximations, inherently contain the non-linear interaction of the source and the filter.

Due to the noise as well as the incompatibility of the sound pressure level meter with the magnetic field of the MRI system, it was not possible to perform a qualitatively sufficient and calibrated audio recording during the MRI examination. Audio and EGG recording were thus performed in a second measurement with careful microphone calibration. Slight changes in the morphometry of the vocal tract for these serial measurements could therefore lead to changes in the vocal tract transfer characteristics. That would further lead to changes in the reactance of this cavity, the resonance frequencies, the coupling to the glottis, and–most importantly–the sound intensities. In order to reduce these factors as much as possible, the measurements were performed on a professional singer, who has practiced the uniform execution of the sound qualities according to Estill Voice Training^®^ for years and has reached the highest training level. In addition, the recording conditions regarding the position (supine), task (same order) and spectrography as online feedback were made as uniform as possible. However, the high accordance of *F*1 derived with inverse filtering of the audio data and *f*_*R*1_ derived from MRI data supports the assumption that the sound production was very uniform. However, a direct comparison of the inverse filtered formant frequency and the numerically determined resonance frequency is not thoroughly possible (Whalen et al., 2022). Additionally auditory masking due to the Lombard effect including respective effects on voice production must be taken into account (for an actual literature review see Nusseck et al. (2022)). Therefore, the singer had time to practice the voice quality without MRI noise. When she was content with the quality, the recording was started approximately one second after the onset of phonation to reduce the Lombard effect as much as possible. Nevertheless, it cannot be completely excluded that the voice production mechanisms were influenced by the measurement conditions. Our working group revealed that also professionally trained singers show systematical effects of body positions leading to changes in respiratory kinematics (Traser et al., 2021b), vertical laryngeal position and vocal tract configuration (Traser et al., 2013). Thus, even if singers have been trained to accurately position the vocal tract and larynx during their Estill Voice Training^®^ education, there may be gravity-related differences between upright and supine phonation. However, since these were systematic differences in singers, and since we performed all the recordings compared here in the supine position, the validity of the data should not be limited.

This study presents data of one professional singer, trained according to one specific vocal school–Estill Voice Training^®^. Extrapolation of the data to other individuals, singers of different sex, or singing techniques is limited. Still, our results show a high agreement with former studies (e.g., the original considerations of Jo Estill and other work on Belting or Western-style classical singing, respectively) regarding resonance properties of the vocal tract or laryngeal function. The analyzed voice qualities offer one variant according to the definition of Estill Voice Training^®^. But, our modeling approach, combined with the signal processing strategy, can be adopted for various variations of voice production. Future work with a larger cohort should clarify whether our results are transferrable to other similarly trained singers or whether our findings regarding voice efficiency are a participant-related phenomenon.

### 4.6 Outlook for future research

The presented data underline the remarkable malleability and variability of the human resonance cavity and the vocal fold vibrations during voice production. It further underlines that these voice features can be trained independently and achieved by one singer. But they also indicate how differently the “instrument voice” operates in various aspects of voice efficiency. It is planned to analyse these aspects in a lager cohort comparing male and female singers. Here acoustically differentiation of the voice qualities by trained listeners would be of interest. Additionally, the influence of acoustic efficiency on the mechanical stress in the vocal folds must further be investigated. In future studies, one should also focus on how a high sound intensity may be responsible for secondary organic voice disorders (Dejonckere and Kob, 2009; Horáček et al., 2009).This study shows the applicability of 3D MRI to obtain individual volumes of the vocal tract, which could be beneficial in teaching and training of voice. Especially singers who choose Estill Voice Training^®^ as a training method are used to work with feedback from a spectrogram. This procedure might benefit from simultaneous acquisition and analysis of EGG-data until sufficient proprioceptive or auditory feedback has been developed by the singer.

### 4.7 Conclusion

This study investigated resonance strategies of different voice qualities and their optimal utilization of the voice source energy. For resonance properties, the data suggests for the assignment in three sub-groups:

For the classical “inverted-megaphone shaped” configuration (first sub-group), an increase in sound pressure level by managing the volume of the hypopharynx and epilarynx tube seems reasonable. We further postulate that narrowing the side cavities decreases the unfavorable antiresonances and increases the bundling of *f*_*R*3_ to *f*_*R*5_ in the frequency range around 3 kHz.

An adjustment of *f*_*R*1_ to *f*_*o*_ seems reasonable for higher fundamental frequencies. In this context, a more constricted hypopharyngeal adjustment improves the acoustic power transfer from the glottis into the vocal tract reflected in the computed lower sound intensity. The vocal fold oscillation was characterized by a medium-strong contact, with the voice source for Opera exhibiting a lower spectral slope (possibly reflecting Level 1 source-filter interactions), which can also be considered more efficient.

For CCM style “megaphone” shaped configuration (second sub-group), amplifying 2*f*_*o*_ by placing *f*_*R*1_ in its high inertance region, in conjunction with a little spectral slope in the particle velocity at the glottis, seems to be an effective strategy in this genre. Impedance matching of the glottal source and the vocal tract, represented by an increased constriction of the epilarynx tube for Belting with an–compared to twang–increased index of contacting, is well reflected in our data.

“Neutral” vocal tract shaping (third sub-group) was not associated with any tuning or high pitch resonance increase. Thus, we cannot expect a significant increase in sound pressure level. Still, power transfer of the source was effective for Speech too. That seems reasonable since it represents the most common voice quality in everyday life without expecting a particular high sound pressure level.

Our findings suggest that Twang, Falsetto, or Sobbing are less efficient concerning energy transfer from the glottis to the vocal tract, their voice source, and their acoustic properties. Energy transfer in Speech was beneficial, but no pronunciation of higher frequencies by specific resonance strategies would reduce the assertiveness with background noise. Thus, these voice qualities are not very loud or predominant to instrumental sounds in their physiology. However, since their timbres are artistically in great demand, they are often used with a microphone or a soft acoustic setting. That might be detrimental to drowning out an orchestra without technical amplification. Belting and Opera are the most efficient qualities and might be better suitable, e.g., for unamplified singing or noisy environments.

From a pedagogic point of view it is important to point out these aspects out for inexperienced singers and explain that each voice quality has a specific dynamic range based on it’s physiology. Advanced technical equipment, knowledge of voice physiology, and auditory feedback (e.g., in-ear monitoring) can be essential for singers to avoid vocal overload or even the development of vocal problems if they aim for too much volume with less efficient voice qualities.

To the best of our knowledge, this is the first study to assess the voice efficiency of different voice qualities in a single subject. Still, future studies should consider the mechanical impact of the vocal folds and check for the transferability of our single-subject findings to other individuals. The newly introduced measure of sound intensity seems to reflect the effectiveness of voice production in a physically motivated and comprehensive way.

## Data Availability

The datasets presented in this study can be found in online repositories. The names of the repository/repositories and accession number(s) can be found below: https://doi.org/10.5281/zenodo.7228354.
