# Supplementary figures and images for: Voice efficiency for different voice qualities combining experimentally derived sound signals and numerical modeling of the vocal tract
Source: Front Physiol. 2022 Dec 23;13:1081622. doi: 10.3389/fphys.2022.1081622 (PMC9822708; doi:10.3389/fphys.2022.1081622)

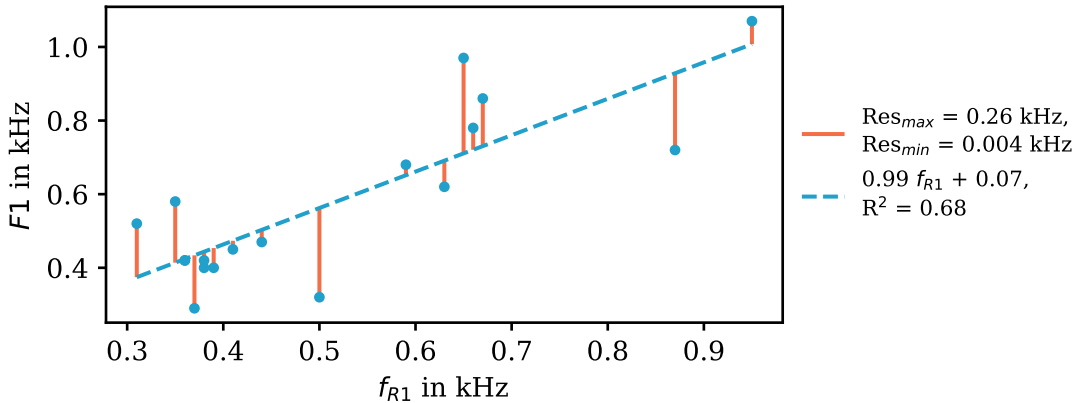

Supplement: Supplementary file 1 [file DataSheet1.ZIP › Fleischer_Suppl_regression.pdf]

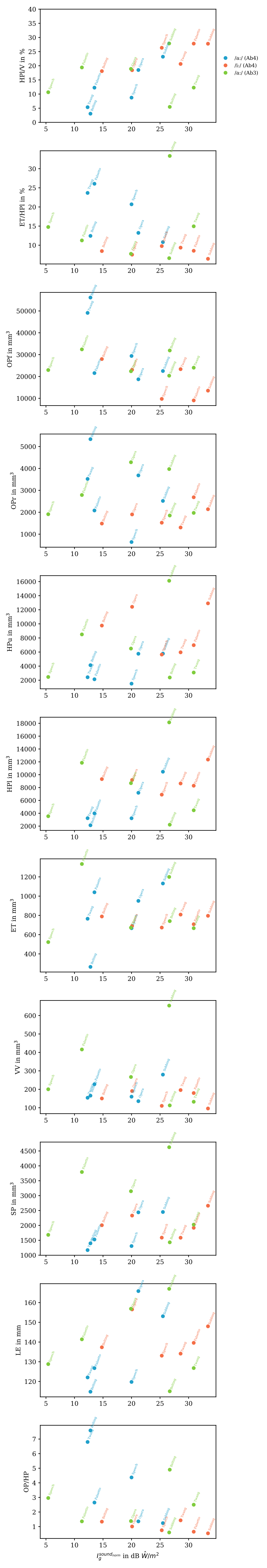

Supplement: Supplementary file 1 [file DataSheet1.ZIP › Fleischer_Suppl_volumes.pdf]
